# Dazhu Hongjingtian (Herba Rhodiolae) for unstable angina pectoris: Protocol for a systematic review and meta-analysis

**DOI:** 10.1097/MD.0000000000013481

**Published:** 2018-12-10

**Authors:** Yu Fan, Xuyu Gu, Heng Zhang

**Affiliations:** a Institute of Molecular Biology and Translational Medicine, The Affiliated People's Hospital, Jiangsu University, Zhenjiang; b Department of General Surgery, Nanjing Lishui District People's Hospital, Nanjing, Jiangsu, China.

**Keywords:** blood rheology, hongjingtian/herba rhodiolae, meta-analysis, systematic review, unstable angina pectoris

## Abstract

**Background:**

Dazhu Hongjingtian (DZHJT), also named Herba Rhodiolae, has been frequently introduced for patients with angina pectoris in China. However, the add-on effect of DZHJT in unstable angina pectoris (UAP) has not been systematically evaluated. The purpose of this protocol is to provide the methods used to assess the efficacy and safety of DZHJT as adjuvant therapy for management of UAP.

**Methods:**

We will extensively search for eligible studies in PubMed, Emase, Cochrane Library, Chinese Biomedical Literature Database, China National Knowledge Infrastructure, Wanfang, and VIP databases up to October 2018. Only randomized controlled trials comparing DZHJT in combination with Western medicine vs Western medicine alone were selected.

The primary outcomes are above 50% reduction in frequency of angina attacks and weekly frequency of angina attacks reduction. The secondary outcomes are the blood rheology parameters (whole-blood viscosity, plasma viscosity, and fibrinogen) and adverse events. We will use RevMan V.5.0 software to perform meta-analysis.

**Results:**

The pooled results will provide a high-quality of evidence of DZHJT as adjuvant therapy in patients with UAP.

**Conclusion:**

This systematic review and meta-analysis will provide up-to-date evidence to evaluate DZHJT as adjuvant therapy in patients with UAP.

**PROSPERO registration number:**

PROSPERO CRD42018111885.

## 1 Introduction

Angina pectoris is chest pain or pressure, usually due to not enough blood flow to the heart muscle. It is generally divided into stable angina pectoris and unstable angina pectoris (UAP). UAP is characterized by attack at rest, usually lasting more than 10 minutes, distinctly more severe, prolonged, or frequent than before.^[1]^ UAP is associated with higher risk of acute myocardial infarction and sudden death. Currently, the common therapeutic strategy includes anti-ischemia, anti-thrombosis, anti-platelet, or revascularization procedures.^[2,3]^

Dazhu Hongjingtian/Herba Rhodiolae (DZHJT) has been widely applied in the management of angina pectoris in China.^[4]^ DZHJT belongs to the species from the family Crassulaceae in the genus *Rhodiola*. The reported pharmacologic actions of DZHJT included cardiac vessels dilation, reduced myocardial oxygen consumption,^[5]^ anti-inflammatory,^[6]^ antidiabetict,^[7]^ and sedative-hypnotic property.^[8]^ A number of studies have assessed the DZHJT as adjuvant therapy in the management of patients with UAP. In this study, we will conduct a systematic review and meta-analysis to evaluate the add-on effects of DZHJT for UAP by analyzing the available randomized controlled trial (RCT).

## 2 Methods

This study will follow the Preferred Reporting Items for Systematic Reviews and Meta-Analyses (PRISMA) guidelines.^[9]^ Ethical approval is not needed because this study only analyzed the study level data.

### 2.1 Inclusion criteria for study selection

#### 2.1.1 Types of studies

All available RCT regarding DZHJT for the treatment of UAP.

#### 2.1.2 Types of patients

Adult patients who are clinically diagnosed with UAP based on the guideline of the Chinese Society of Cardiology,^[10]^ American College of Cardiology Foundation/American Heart Association,^[11]^ World Health Organization,^[12]^ or European Society of Cardiology.^[13]^ Patients with stable coronary heart disease or acute myocardial infarction are excluded.

#### 2.1.3 Types of interventions

The experimental group receives DZHJT in combination of conventional Western medicine, while the control group receives the same Western medicine.

#### 2.1.4 Types of outcome measures

The primary outcomes are above 50% reduction in frequency of angina attacks and weekly frequency of angina attacks reduction. The secondary outcomes are the changes of whole-blood viscosity, plasma viscosity, or fibrinogen and adverse events.

### 2.2 Data source and search strategy

#### 2.2.1 Electronic searches

We will systematically PubMed, Emase, Cochrane Library, Chinese Biomedical Literature Database, China National Knowledge Infrastructure, Wanfang, and VIP databases up to October 1, 2018. Only articles that published in Chinese and English were included.

#### 2.2.2 Search strategy

Search keywords included “acute coronary syndrome” OR “unstable angina pectoris” OR “angina” OR “UAP” AND “rhodiola” OR “hong jing tian” OR “hongjingtian” AND “randomized” OR “randomised” AND “random”.

#### 2.2.3 Searching for other resources

A manual search will carry out from the reference lists of included studies and relevant reviews. The clinical trial registry at https://clinicaltrials.gov/ will also retrieve unpublished protocols and summary results. If required data are not reported in included studies, the authors will contact the corresponding author through e-mail for additional information.

### 2.3 Study selection and data extraction

Two authors will independently assess the potential eligible studies based on our predefined inclusion criteria and then extract relevant data from the included studies. Any disagreements in this process were resolved by discussion. The study selection process will be presented according to PRISMA flowchart. Data extracted will include the surname of the first author, publication year, number of patients, mean age or age range, percentage of men, diagnostic criteria, dose of DZHJT, duration of treatment, types of outcome measure, methodologic data, and Traditional Chinese Medicine syndromes.

### 2.4 Risk of bias

Cochrane Collaboration tool will be used to the assessment of risk of bias. The items assessed the methodologic quality include random sequence generation, allocation concealment, blinding of participants and personnel, blinding of outcome assessment, incomplete outcome data, selective reporting, and other bias. Each study will be classified as “high,” “ low,’, or “ unclear ” risk of bias.

### 2.5 Data synthesis and analysis

#### 2.5.1 Measures of treatment effect

RevMan 5.0 software and STATA 12.0 software are planned to perform the meta-analysis. For dichotomous data, the pooled results will be summarized as a rate ratio with 95% confidence intervals (CIs). For continuous data, results will be presented as the mean difference (MD) with 95% CI. A *P*-value <.05 is considered statistically significant.

#### 2.5.2 Assessment of heterogeneity

The Cochrane *Q* statistic and *I*^2^ index will apply to assess the heterogeneity across the studies. We will consider the significant heterogeneity when *I*^2^ value is over 50% and/or Cochrane Q statistic test <0.10. A random effect meta-analysis will apply in the presence of significant heterogeneity; otherwise we will use a fixed-effect model.

#### 2.5.3 Assessment of publication bias

We will construct a funnel plot to evaluate the potential publication bias when there was sufficient number of studies (at least 10 studies). Begg rank correlation test and Egger linear regression test will also be used to quantitatively assess the publication bias.

#### 2.5.4 Subgroup analysis

Subgroup analysis will be conducted to assess the heterogeneity. We will explore the heterogeneity by dosage of DZHJT, treatment duration, type of diagnostic criteria, and study quality.

#### 2.5.5 Sensitivity analysis

A leave-one-out sensitivity analysis will perform to test the stability of the pooling results.

## 3 Discussion

This protocol will summarize the up-to-date data to evaluate the efficacy and safety of DZHJT as adjuvant therapy in patients with UAP. The findings of this study will provide evidence whether DZHJT combined with Western medicine will achieve additional benefits in patients with UAP. Nevertheless, the safety of adjuvant treatment with DZHJT will also be evaluated. Moreover, the strength of the findings will be summarized by GRADE quality of evidence. Findings of this systematic review and meta-analysis will help clinicians make decisions in clinical practice and overcome the methodologic quality of future studies.

## Author contributions

**Conceptualization:** Heng Zhang.

**Data curation:** Yu Fan, Xuyu Gu.

**Formal analysis:** Yu Fan, Xuyu Gu.

**Investigation:** Yu Fan, Xuyu Gu.

**Methodology:** Yu Fan.

**Project administration:** Heng Zhang.

**Writing - original draft:** Xuyu Gu.

**Writing - review & editing:** Heng Zhang.
